# Intranodal Epithelioid Schwannoma in an Intraparotid Lymph Node: A Case Report and Literature Review

**DOI:** 10.1002/ccr3.73709

**Published:** 2026-10-12

**Authors:** Maryam Vajihinejad, Kianoosh Najafi, Mohammad Hossein Ahrar, Ahmad Shamsi

**Affiliations:** ^1^ Department of Pathology, Shahid Sadoughi Hospital Shahid Sadoughi University of Medical Sciences Yazd Iran; ^2^ School of Medicine and Surgery University of Naples Federico II Naples Italy; ^3^ Department of Radiology, Shahid Sadoughi Hospital Shahid Sadoughi University of Medical Sciences Yazd Iran; ^4^ Student Research Committee Shahid Sadoughi University of Medical Sciences Yazd Iran

**Keywords:** immunohistochemistry, lymph nodes, parotid gland, peripheral nerve sheath neoplasms, schwannoma

## Abstract

An epithelioid schwannoma arising within an intraparotid lymph node is a rare benign lesion that may mimic metastatic melanoma, metastatic carcinoma, or a primary salivary gland neoplasm. Recognizing the intranodal architecture and integrating morphology with an appropriate immunohistochemical panel are essential to avoid misclassification and unnecessary aggressive oncological management.

## 1 Introduction

Schwannomas, also known as neurilemmomas, are benign, slow‐growing, encapsulated tumors that originate from Schwann cells of the peripheral nerve sheath. These neoplasms can occur at any anatomical site, with a predilection for the head, neck, and flexor surfaces of the extremities [1, 2, 3]. While schwannomas are relatively common peripheral nerve sheath tumors, their occurrence within the parenchyma of a lymph node or intranodal schwannoma is an exceptionally rare phenomenon [4]. The majority of schwannomas are solitary and sporadic lesions; however, the intranodal variant presents a unique diagnostic challenge due to its unexpected location and the broad differential diagnosis of spindle cell lesions within a lymph node [2, 4, 5].

Given the extreme rarity of this entity, we present the following case to contribute to the understanding of the clinical and pathological features of intranodal schwannoma in the parotid salivary gland, with emphasis on its diagnostic challenges and management implications.

## 2 Case History/Examination

A 40‐year‐old woman, with no significant medical history, presented with a firm, mobile, slowly enlarging mass in the right preauricular region, causing unilateral facial swelling. The mass had been first noted 6 months earlier and had become tender 10 days before presentation. On physical examination, a palpable mass measuring approximately 30 × 20 mm was noted.

Contrast‐enhanced computed tomography (CT) images of the neck demonstrated a well‐circumscribed mass measuring 27 × 18 mm in the superficial lobe of the right parotid gland. The lesion showed marked enhancement and a central low‐attenuation area, with smooth margins and preservation of the adjacent fat planes. No evidence of adjacent osseous destruction or regional lymphadenopathy was identified (Figure 1).

**FIGURE 1:**
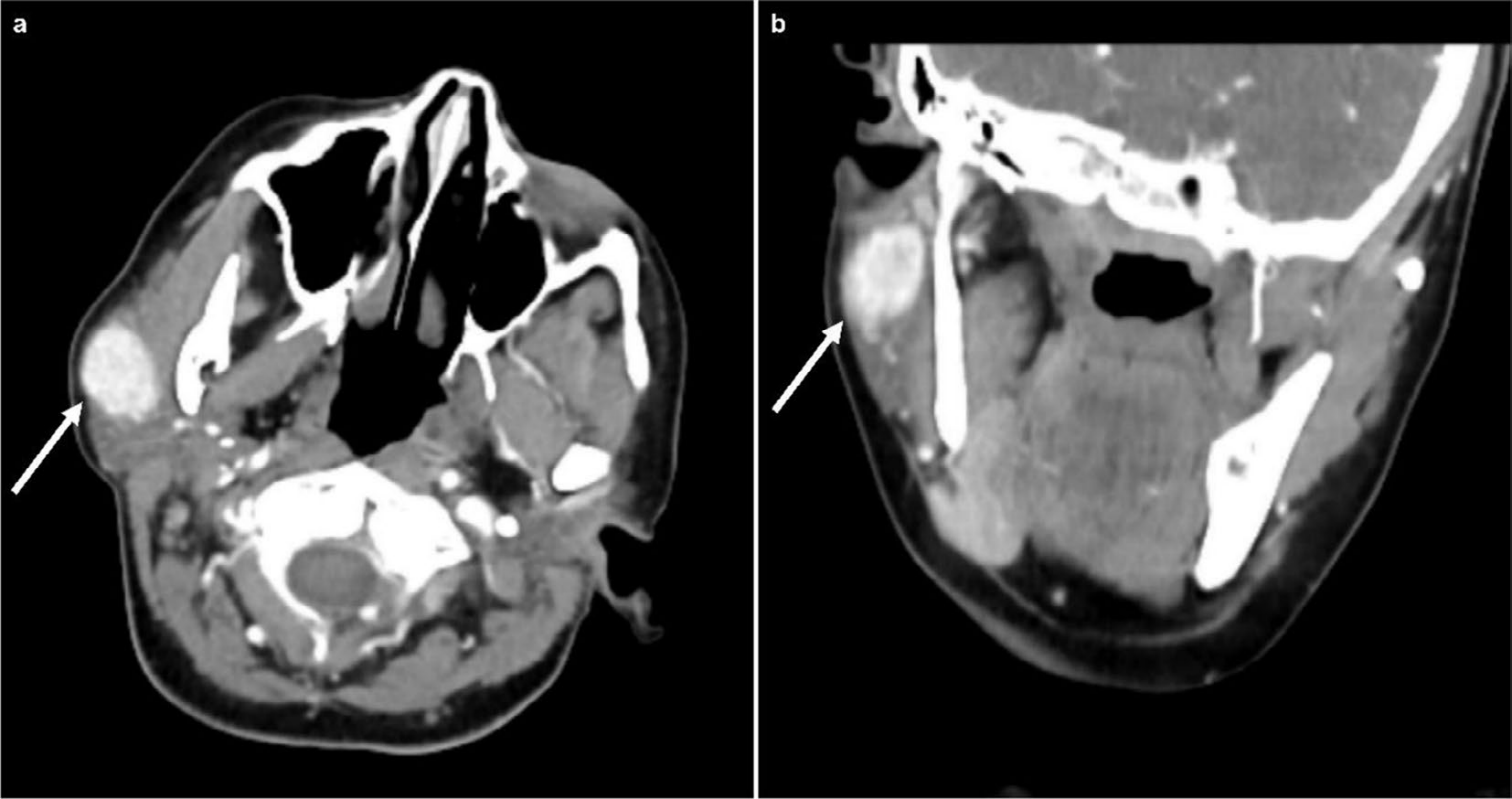
Contrast‐enhanced CT images of the neck demonstrating a well‐circumscribed mass in the superficial lobe of the right parotid gland: (a) axial image and (b) coronal image.

## 3 Methods (Differential Diagnosis, Investigations, and Treatment)

Prior to CT evaluation, ultrasonography demonstrated a relatively round, well‐defined hypoechoic mass in the right parotid region. Fine‐needle aspiration and core‐needle biopsy were not performed. The preoperative differential diagnosis included primary parotid neoplasms, intraparotid lymph node involvement, nerve sheath tumors, and metastatic lesions; however, a definitive preoperative diagnosis was not established.

The patient subsequently underwent superficial parotidectomy with preservation of the facial nerve. Given the lesion's location in the superficial parotid region, the preoperative differential diagnostic considerations, and the uncertainty regarding its exact origin, superficial parotidectomy was selected to achieve complete excision while allowing preservation and assessment of the facial nerve. Local excision was not selected because the exact origin of the mass was uncertain preoperatively. Intraoperatively, the mass was not associated with a recognizable nerve and did not appear to arise from the facial nerve or any other identifiable nerve. The facial nerve was preserved, and postoperative clinical assessment demonstrated intact facial nerve function.

Gross examination revealed a well‐circumscribed, encapsulated tumor measuring 27 × 18 mm. The cut surface was gray‐brown and surrounded by a thin rim of unremarkable brown parotid gland tissue. Formalin‐fixed, paraffin‐embedded (FFPE) tissue sections were cut at 4 μm and stained with hematoxylin and eosin (H&E) using standard protocols. Histologic examination demonstrated a well‐circumscribed, encapsulated neoplasm composed predominantly of epithelioid cells, entirely contained within an intraparotid lymph node (Figure 2a,b). At low magnification, the lesion was located within the lymph node, with the lymph node capsule and subcapsular sinus identifiable at the periphery and adjacent parotid gland tissue present outside the nodal capsule (Figure 2a). A peripheral rim of residual lymphoid tissue was present around the tumor (Figure 2b).

**FIGURE 2:**
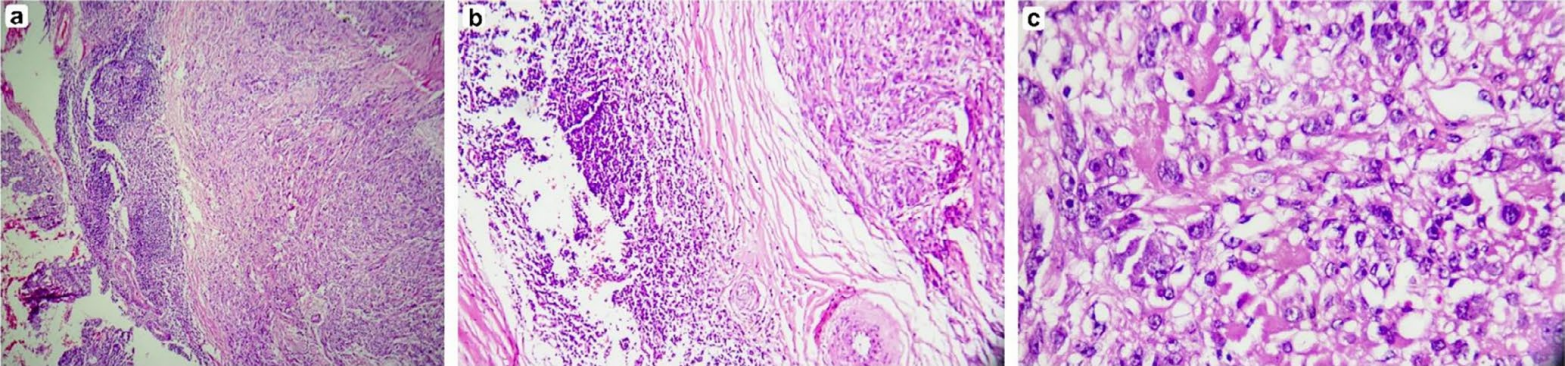
Histopathological findings of intranodal epithelioid schwannoma. (a) Low‐power view demonstrating a well‐circumscribed, encapsulated tumor entirely contained within an intraparotid lymph node, with adjacent parotid gland tissue outside the lymph node capsule (H&E, ×4). (b) Intermediate‐power view showing the lymph node capsule, subcapsular space, residual lymphoid tissue, and the well‐circumscribed encapsulated epithelioid schwannoma (H&E, ×10). (c) High‐power view demonstrating the predominantly spindle‐to‐epithelioid cell and abundant eosinophilic cytoplasm (H&E, ×40).

The neoplasm was composed predominantly of sheets of epithelioid cells within a hyalinized stroma, with focal areas showing conventional spindle‐cell morphology. The tumor cells exhibited abundant eosinophilic cytoplasm and round‐to‐oval nuclei with prominent nucleoli. No significant cytologic atypia, increased mitotic activity, tumor necrosis, or extension beyond the lymph node was identified. At higher magnification, the epithelioid tumor cells were polygonal, with abundant eosinophilic cytoplasm and round‐to‐oval nuclei (Figure 2c).

Immunohistochemical studies showed diffuse, strong positivity for S100 protein and vimentin in the tumor cells. The neoplastic cells were negative for myogenic markers (desmin, myogenin, and SMA), melanocytic markers (HMB‐45 and Melan‐A), CD34, lymphoid markers (CD45, PAX5, CD23, and CD30), and epithelial/perineurial markers (pan‐cytokeratin, CK AE1/AE3, and EMA). SMA expression was absent in the tumor cells, while vascular smooth muscle cells served as an internal positive control. The Ki‐67 labeling index was < 1%, indicating a low proliferative index. Taken together, the histomorphological and immunohistochemical findings supported the diagnosis of epithelioid intranodal schwannoma (Figure 3). Because immunohistochemical studies were performed approximately 2 years ago, the original technical records containing antibody clone and kit information are no longer retrievable from the laboratory system. Therefore, these details could not be reliably reported retrospectively. Vascular smooth muscle cells served as an internal positive control for SMA; other antibody‐specific internal or external control details were not retrievable from the original records.

**FIGURE 3:**
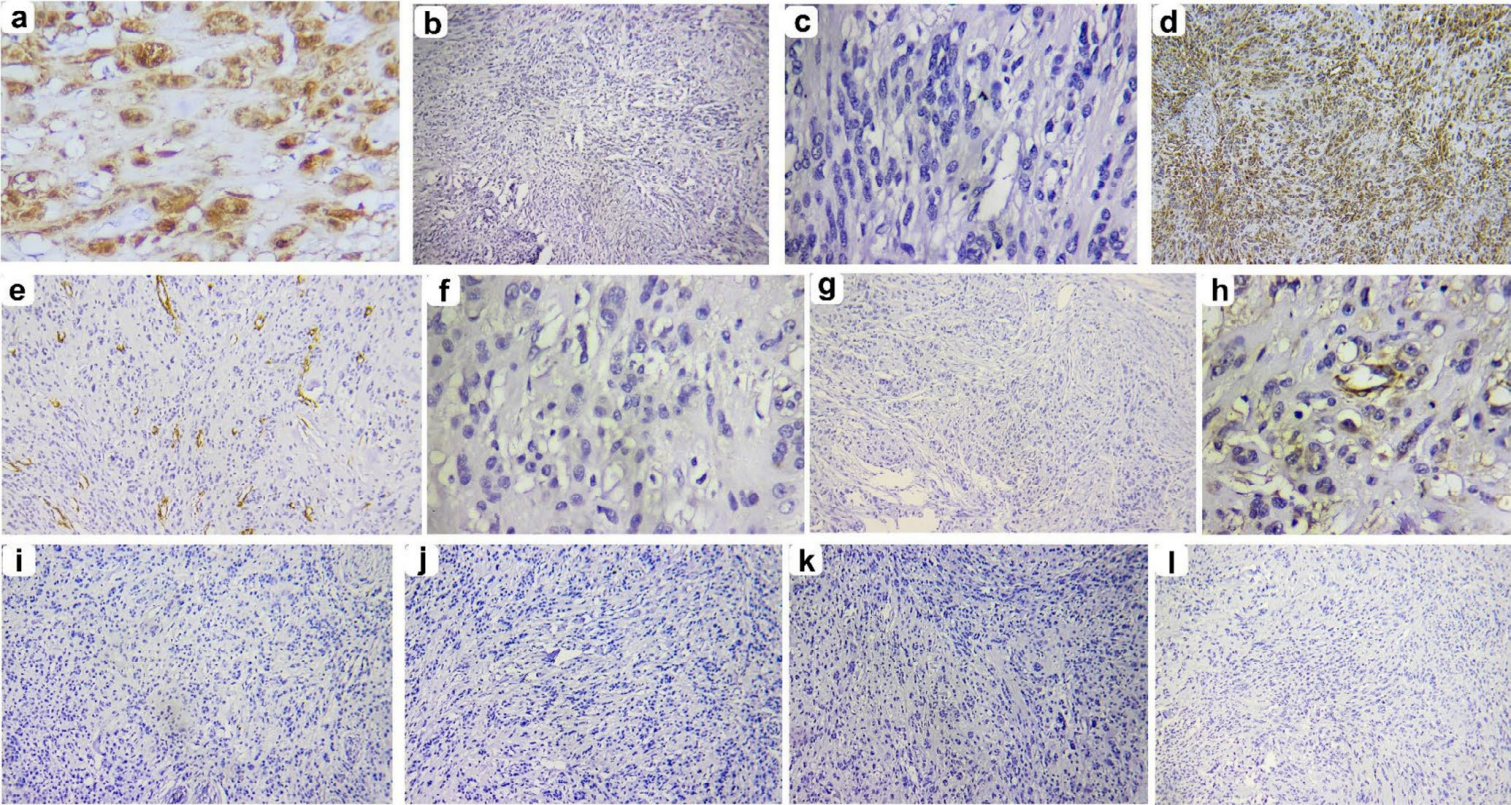
Immunohistochemical studies of the intranodal epithelioid schwannoma. (a) S100, ×40, strong and diffuse nuclear and cytoplasmic immunoreactivity in the neoplastic cells. (b) CD30, ×10, absence of staining in the neoplastic cells. (c) PAX5, ×40, no immunoreactivity in the neoplastic cells. (d) Vimentin, ×10, diffuse cytoplasmic immunoreactivity in the neoplastic cells. (e) SMA, ×10, absence of staining in the neoplastic cells, with retained staining in the vascular smooth muscle walls as an internal positive control. (f) Melan‐A, ×40, no immunoreactivity in the neoplastic cells. (g) Ki‐67, ×10, very low proliferative index, with nuclear labeling in < 1% of neoplastic cells. (h) CD34, ×40, absence of staining in the neoplastic cells, with positive staining in endothelial cells. (i) CD45, ×10, absence of CD45 expression in the neoplastic cells. (j) EMA, ×10, no immunoreactivity in the neoplastic cells. (k) HMB‐45, ×10, no immunoreactivity in the neoplastic cells. (l) Pan‐cytokeratin, ×10, negative staining in the neoplastic cells.

The differential diagnosis of an epithelioid lesion arising within an intraparotid lymph node is broad. It includes metastatic malignant melanoma, metastatic carcinoma, myoepithelial neoplasms, epithelioid malignant peripheral nerve sheath tumor (MPNST), perineurioma, and less commonly, lymphoid and fibroblastic tumors. Among these entities, metastatic melanoma and metastatic carcinoma represent the most important diagnostic considerations due to their frequent involvement of intraparotid lymph nodes and their significant morphologic overlap with epithelioid schwannoma. Detailed histopathologic evaluation is essential for accurate diagnosis, including assessment of growth pattern, cytologic atypia, mitotic activity, necrosis, and residual lymphoid tissue (Table 1). In the present case, the combined morphological and immunohistochemical findings supported Schwannian differentiation and argued against the principal epithelial, melanocytic, myogenic, lymphoid, and perineurial mimics. In addition, the patient had undergone clinical evaluation for potential cutaneous, mucosal, and ocular primary melanoma, and no evidence of a primary melanocytic lesion was identified.

**TABLE 1:** Differential diagnosis of an epithelioid lesion arising within an intraparotid lymph node, their immunohistochemical findings, histologic, and distinguishing features.

Diagnosis	Histologic features	Key IHC findings	Distinguishing features
Metastatic malignant melanoma	Epithelioid or spindle cells with variable cytological atypia; prominent nucleoli, mitotic activity, necrosis, and/or melanin pigment may be present	S100 and SOX10 are usually positive; HMB‐45 and Melan‐A are often positive; PRAME may be positive	Major diagnostic mimic in an intraparotid lymph node; conventional melanocytic markers may occasionally be absent; clinical evaluation for a cutaneous, mucosal, or ocular primary is important
Metastatic carcinoma	Glands, nests, trabeculae, or sheets of malignant epithelial cells; may replace or efface nodal architecture	Pan‐CK/CK AE1/AE3 and often EMA positive	Epithelial differentiation and metastatic nodal involvement; clinical and radiological evidence of primary carcinoma may be present
Epithelioid malignant peripheral nerve sheath tumor	Epithelioid and/or spindle cells with cytological atypia, infiltrative growth, increased mitotic activity, and possible necrosis	S100 and SOX10 may be positive but are often focal or reduced; INI1/SMARCB1 loss may occur; Ki‐67 is often elevated	Malignant cytological and architectural features; infiltrative borders and increased proliferation; may be associated with a nerve, NF1, or prior radiation
Myoepithelial neoplasm	Epithelioid, spindle, or plasmacytoid cells arranged in nests, cords, or sheets within myxoid or hyalinized stroma	S100 and SOX10 are often positive; variable cytokeratin, EMA, p63, and calponin expression	Co‐expression of epithelial and myoepithelial markers; salivary gland or soft‐tissue myoepithelial phenotype
Perineurioma	Bland spindle‐to‐epithelioid cells with whorled, storiform, or fascicular architecture	EMA, Claudin‐1, and/or GLUT1 positive; S100 usually negative or only focal	Perineurial rather than Schwannian differentiation; typically low proliferative activity and absence of necrosis
Lymphoid neoplasms	Diffuse, nodular, or follicular proliferation of lymphoid cells; nodal architecture may be effaced	CD45 positive with lineage‐specific markers	Hematolymphoid phenotype and characteristic lymphoid architecture
Vascular lesions	Vascular channels or epithelioid/spindle endothelial proliferation	CD31 and ERG are typically positive; CD34 may be positive	Endothelial differentiation and characteristic vascular architecture
Fibroblastic neoplasms	Spindle or epithelioid proliferation in collagenous or myxoid stroma; variable cellularity	Usually S100 and SOX10 negative; CD34 and/or SMA may be variably expressed	Lack of Schwannian differentiation and fibroblastic/myofibroblastic phenotype
Myogenic neoplasms	Fascicular or sheet‐like proliferation of spindle or epithelioid cells; variable cytological atypia	SMA, desmin, and/or other myogenic markers may be positive; myogenin may be expressed in skeletal muscle differentiation	Myogenic differentiation and appropriate clinical/pathological context
Intranodal epithelioid schwannoma	Well‐circumscribed and usually encapsulated; nests, cords, and sheets of predominantly epithelioid cells with abundant eosinophilic cytoplasm in a hyalinized or collagenous stroma; minimal cytological atypia; mitoses and necrosis typically absent or rare	Diffuse, strong S100 and SOX10 expression; vimentin positive; melanocytic, epithelial, myogenic, and perineurial markers generally negative; low Ki‐67 index; INI1/SMARCB1 typically retained	Schwannian differentiation with a benign cytomorphologic profile and well‐circumscribed intranodal growth; absence of destructive invasion, significant atypia, necrosis, or increased proliferative activity

Abbreviations: CD, cluster of differentiation; CK, cytokeratin; EMA, epithelial membrane antigen; ERG, ETS‐related gene; GLUT1, glucose transporter 1; HMB45, human melanoma black 45; IHC, immunohistochemistry; INI1 (SMARCB1), integrase interactor 1; Ki‐67, Ki‐67 proliferation index, NF1; Neurofibromatosis Type 1; p63, tumor protein p63; Pan‐CK, pancytokeratin; PRAME, preferentially expressed antigen in melanoma; SMA, smooth muscle actin; SOX10, SRY‐box transcription factor 10.

## 4 Outcome and Follow‐Up

Following complete surgical excision, the postoperative course was uneventful. At the two‐year follow‐up, physical examination revealed no palpable abnormality, and ultrasonographic evaluation of the parotid region demonstrated no evidence of local recurrence. The patient remained completely asymptomatic and reported no specific complaints, including facial nerve dysfunction, pain, or local discomfort. Facial nerve function was assessed clinically before surgery, after surgery, and at the follow‐up examination and remained intact.

## 5 Discussion

Schwannomas are benign tumors that develop from Schwann cells of the peripheral nerve sheath. Primary schwannomas developing inside lymph nodes (intranodal schwannomas) are very rare, although schwannomas often develop in the head and neck region [1]. In the parotid region, lesions can often be confused with salivary gland and facial nerve neoplasms or lymph node metastases, which complicates the diagnosis [6, 7]. This case is of interest because it is located intranodally inside an intraparotid lymph node, and because it has epithelioid morphology. This case highlights the diagnostic challenges posed by an intranodal lesion with predominantly epithelioid morphology and emphasizes the importance of integrating histopathological and immunohistochemical findings to avoid misclassification and unnecessary aggressive management.

To assess the rarity and novelty of this entity, a structured literature search was conducted in PubMed, Scopus, and Web of Science from database inception through September 8, 2026, without date restrictions. Combinations of the terms “intranodal schwannoma,” “intraparotid lymph node,” “parotid lymph node,” “schwannoma,” “neurilemmoma,” and “epithelioid schwannoma” were used. Only English‐language publications were considered. Cases were included when the available clinical, surgical, and/or histopathological information supported a true intranodal origin, whereas schwannomas arising from the facial nerve, parotid parenchyma, or other nerves in the parotid region without evidence of intranodal origin were excluded. Based on this structured search, and to the best of our knowledge, the present case may represent the second reported intraparotid intranodal schwannoma and the first reported case with predominantly epithelioid morphology.

Intranodal schwannomas of the head and neck region are summarized in Table 2. Black et al., Silvestre et al., Mirea et al., and Hussain et al. reported intranodal schwannoma in the cervical region [1, 2, 4, 9]. Grant et al. described the first case of intranodal schwannoma located in the parotid gland with ancient neurilemmoma histopathology [8]. These reports demonstrate a wide range of patient ages and cervical lymph node locations, with most patients presenting with a painless, slowly enlarging mass or as an incidental finding. The reported tumors ranged from 15 × 10 × 10 mm to 110 × 80 mm and were predominantly conventional or classical schwannomas, with occasional variants including ancient and cellular schwannoma. Imaging findings were variable and generally nonspecific, reflecting the difficulty of establishing a definitive diagnosis preoperatively. Immunohistochemically, most reported lesions showed S100 positivity, with variable expression of other neural or mesenchymal markers and generally negative muscle, epithelial, and melanocytic markers. Surgical resection was the main treatment in all reported cases. Follow‐up information was limited, although most cases showed no recurrence; notably, one recent case developed malignant transformation to MPNST during follow‐up.

**TABLE 2:** Literature review of cervical intranodal schwannoma cases.

References	Year	Age	Sex	Anatomical location	Tumor size	Clinical presentation	Radiological findings	Histological subtype	Principal IHC finding	Treatment	Duration of follow‐up	Outcome
Grant et al. [8]	2009	67 years	F	Superficial part of Lt parotid	42 × 28 × 19 mm	6 months, slowly enlarging, non‐tender ovoid mass, well‐circumscribed and mobile	Lobulated, intermediate‐signal mass on MRI	Ancient neurilemmoma	Positive S100 and glial fibrilla acidic protein; negative for desmin, SMA, epithelial markers, CD68, CD117, synaptophysin, and HMB‐45	Surgical resection	8 months	No local recurrence
Black et al. [4]	2010	77 years	F	Lt cervical	15 × 10 × 10 mm	A painless, slowly growing mass	Not reported	Conventional (classical) schwannoma	Positive S100 and Fascin‐1; focally positive CD146; negative pan‐melanoma cocktail, cytokeratin cocktail, desmin, SMA, EMA, CD21, CD23, CD35, and CD45	Surgical resection	Not reported	No recurrence
Silvestre et al. [2]	2019	69 years	F	Rt cervical	66 × 57 × 38 mm	Asymptomatic, incidental finding during thyroidectomy totalization; bilateral sensorineural hypoacusia	Hypoechoic nodule on ultrasonography. Mildly enhancing nodule on CT scan.	Conventional (classical) schwannoma	Positive S100, SOX10, and nestin; negative SMA, desmin, CD34, HMB‐45, and Melan‐A; Ki‐67 < 1%	Surgical resection (Thyroidectomy totalization with excision of the mass)	Not reported	Postoperative right vocal cord paralysis with incomplete glottic closure; no recurrence reported
Mirea et al. [1]	2022	50 years	F	Rt. lateral cervical	45 × 25 mm	1‐year painless, slowly growing swelling	Well‐circumscribed, homogeneous mass and modest contrast enhancement on CT scan	Conventional (classical) schwannoma	Positive S100; negative SMA and desmin; low Ki‐67 (5%)	Surgical resection	Not reported	Short‐ and long‐term post‐operative evolution was favorable
Hussain et al. [9]	2026	46 years	M	Rt cervical	110 × 80 mm	2 years slowly enlarging, painless and mobile mass	A complex mass on MRI	Cellular schwannoma	Positive S100 and SOX10; negative β‐catenin, Desmin, CD34, and EMA	Surgical resection without complete removal because of adherence and proximity to vital structures	18 months	malignant transformation of a pre‐existing intranodal schwannoma to MPNST

Abbreviations: CT, computed tomography; F, female; IHC, immunohistochemistry; Lt, left; M, male; MPNST, malignant peripheral nerve sheath tumor; MRI, magnetic resonance imaging; Rt, right.

The main histopathological characteristic of intranodal schwannoma is a capsule‐encased tumor of a nerve sheath which is completely encased by the lymph node capsule and is usually surrounded by a layer of compressed residual lymphoid tissue [1]. Although our case showed a few focal areas of classical spindle‐shaped cells which contained nuclear palisading and Verocay bodies, almost 80% of the lesion was made up of epithelioid cells. Compared to classical schwannomas, which are usually characterized by a biphasic pattern of Antoni A and B areas containing prominent Verocay bodies, the epithelioid variant comprises less than 5% of all schwannomas and is a recognized diagnostic challenge due to its close resemblance to a range of melanocytic and epithelial tumors.

The differential diagnosis for an intranodal epithelioid lesion is broad and potentially misleading. Due to its location within a parotid lymph node, the main concerns are metastatic malignant melanoma and metastatic carcinoma. Therefore, exclusion of these malignancies requires a detailed evaluation of cellular atypia, mitotic activity, and growth patterns [10].

For the reported case, Schwannian differentiation was supported primarily by diffuse, strong S100 expression in conjunction with the characteristic morphology. Vimentin expression was also observed but is nonspecific. The absence of HMB‐45 and Melan‐A expression argued against metastatic melanoma, while negative cytokeratin staining argued against metastatic carcinoma and other epithelial neoplasms [11]. However, negative HMB‐45 and Melan‐A staining should be interpreted cautiously, as some melanomas may lack expression of conventional melanocytic markers. In the present case, clinical evaluation for a potential cutaneous, mucosal, or ocular primary lesion was reportedly negative.

Myoepithelial tumors may express S100 protein as well, but they will show expression of myogenic and myoepithelial markers such as SMA and p63 [12]. The lack of SMA expression supported the absence of myogenic differentiation. Similarly, the absence of CD45, CD30, and PAX5 expression argued against a lymphoid neoplasm, while negative CD34 and EMA staining did not support fibroblastic or perineurial differentiation, respectively. Taken together, the morphological and immunohistochemical findings supported Schwannian differentiation and argued against the principal epithelial, melanocytic, myogenic, lymphoid, fibroblastic, and perineurial mimics.

An important differential diagnosis was epithelioid MPNST. In the present case, several features supported a benign lesion, including its well‐circumscribed and encapsulated growth pattern, bland cytology, absence of significant atypia, absence of increased mitotic activity, absence of tumor necrosis, and lack of extension beyond the lymph node. The Ki‐67 labeling index was < 1%, supporting a low proliferative index, and no recurrence was observed during 2 years of follow‐up. Taken together, these morphological, immunohistochemical, and clinical findings supported the diagnosis of a benign epithelioid schwannoma rather than an epithelioid MPNST. Additional immunohistochemical markers that may be useful in this differential diagnosis, including SOX10 and INI1/SMARCB1, were not performed in the present case and should therefore not be inferred.

## 6 Conclusion

Epithelioid intranodal schwannoma arising within an intraparotid lymph node is an exceptionally rare benign neoplasm that may clinically and radiologically mimic a primary salivary gland tumor or metastatic lymphadenopathy. Because preoperative diagnosis may be challenging, recognition of the intranodal architecture and integration of histopathological findings with an appropriate immunohistochemical panel are essential for accurate diagnosis. Awareness of this uncommon entity may help prevent misclassification and unnecessary aggressive oncological management. Complete surgical excision is generally associated with an excellent prognosis.

## Author Contributions

**Maryam Vajihinejad:** conceptualization, supervision, data curation, writing – review and editing, writing – original draft. **Kianoosh Najafi:** writing – original draft, writing – review and editing. **Mohammad Hossein Ahrar:** writing – original draft, writing – review and editing, data curation. **Ahmad Shamsi:** writing – original draft, writing – review and editing.

## Funding

The authors have nothing to report.

## Ethics Statement

This case report was approved by the Research Ethics Committee of the School of Medicine, Shahid Sadoughi University of Medical Sciences (Approval ID: IR.SSU.MEDICINE.REC.1405.144).

## Consent

Written informed consent was obtained from the patient for publication of the clinical details and accompanying images.

## Conflicts of Interest

The authors declare no conflicts of interest.

## Data Availability

The data that support the findings of this study are available from the corresponding author upon reasonable request.
